# A Systematic Review of 5110 Cases of Monkeypox: What Has Changed Between 1970 and 2022?

**DOI:** 10.7759/cureus.30841

**Published:** 2022-10-29

**Authors:** Rajesh Kumar, Shruti Singh, Sunil K Singh

**Affiliations:** 1 Pharmacology, All India Institute of Medical Sciences, Patna, Patna, IND

**Keywords:** travel-related infection, smallpox vaccine, monkeypox transmission, men who have sex with other men (msm), monkeypox

## Abstract

The recent monkeypox (MPVX) outbreak has been characterized by an unprecedented increase in cases, unlike any other outbreaks in the past. The disease pattern and transmissibility are also different from previous outbreaks. This systematic review aimed to evaluate whether the current outbreak has significant contrasting features from the previous ones, necessitating changes in prevention and control guidelines. A thorough literature search related to MPVX infection was performed on the online databases PubMed, Google Scholar, and ScienceDirect using appropriate keywords "MPVX", "men who have sex with men (MSM)", "transmission", and "smallpox vaccination", for choosing relevant articles from the inception of MPVX in 1970 to August 31, 2022. We identified 5110 cases of MPVX, documented in 63 articles on MPVX. We discovered that the median age of MPVX infection has slowly increased since its inception, and currently, it is more common in adults. Compared to previous outbreaks, a significantly greater male preponderance is witnessed in the current outbreak. Only 238 (4.65%) out of the 5110 evaluated patients were vaccinated with the smallpox vaccine in our review. There were 107 mortalities, most of which were children below the age of 10 years. Out of the 1534 cases identified in 2022, 1134 (73.92%) patients admitted that they had been involved in sexual relations within the last 21 days (MSM/gay/bisexual). We found that in contrast to previous outbreaks, human-to-human transmission is more common in this outbreak, with most cases having no link with endemic countries. There are evolving traits and undetected transmission modes of MPVX infection that require new disease mitigation strategies.

## Introduction and background

The WHO declared monkeypox (MPVX) a Public Health Emergency of International concern on July 23, 2022. As of September 22, 2022, 64,290 cases have been reported (63,711 of these cases are in locations that have not historically reported MPVX), with 20 confirmed deaths. MPVX is caused by a virus belonging to the Poxviridae family, *Chordopoxvirinae* subfamily, and *Orthopoxvirus* genus [1]. It is very similar to the Variola (smallpox) virus. MPVX was discovered in a Danish Laboratory in 1958 as the causative agent of smallpox (SPX)-like illness in Cynomolgus monkeys; hence the name MPVX [2]. Only animals were initially considered susceptible to the virus, but after a confirmed infection in a nine-month-old child in the Democratic Republic of Congo (DRC) in 1970, its zoonotic nature was confirmed [3]. In the beginning, the disease was primarily confined to African countries. However, an outbreak in the United States of America in 2003 heralded the virus’s non-endemic spread [4-7]. The most enormous documented explosion occurred in Nigeria in 2017, almost 40 years after the last case [8]. One case was reported in Israel in 2018 and in Singapore in May 2019. Three patients were reported in the United Kingdom between 2018 and 2019; all of them had a history of recent travel to Nigeria [9-12]. The current outbreak of 2022 began in the United Kingdom in a patient who also had recently traveled to Nigeria in May 2022. Later, two additional cases involving cohabitants of the first case were identified. Within the first month of this outbreak, there were more than 3000 cases reported across 50 countries, many of them non-endemic.

Apart from this multi-country community transmission, which is alarming, the clinical features of the disease have also evolved with a more subclinical prodrome, less extensive skin lesions localized to genital regions, and a well-defined prevalence among the "men who have sex with men" (MSM) community. In most of the earlier outbreaks, the index case had acquired the infection from an animal source. However, this contrasts with the current outbreak, with human-to-human transmission being the norm rather than the exception [13,14]. Between 1980 and 1984, clinical and subclinical diseases mainly occurred in children younger than 10 years of age; however, current trends suggest that the disease is more prevalent among adults. Previously, most of the deaths were seen in children; however, the mortality statistics related to the current outbreak are unclear. One fact consistent across outbreaks is the greater attack rate in individuals not vaccinated against smallpox.

There is still much confusion regarding the epidemiology of the current outbreak and how much it deviates from the historical trend. There are chances of a “misinfodemic” full of confusion about the disease's origin and transmission mode [15,16]. Already, there are myths circulating that MPVX is connected to the coronavirus disease (COVID-19) vaccine (the AstraZeneca vaccine uses a chimpanzee viral vector). One of the most common pieces of misinformation circulating is that MPVX is simply a conspiracy cooked up by various international institutions [17]. More importantly, the recent clustering of cases around sexual networks is driving a sense of stigma that might deter high-risk groups from coming forward and seeking help [18]. We desperately need to understand what is the driving force behind the outbreak. Is clustering at specific events responsible? Why are individuals with high-risk behavior being affected more predominantly? In short, how is the current trend different from the previous ones?

In light of this, this systematic review attempts to compare the pattern of past and current outbreaks and suggest recommendations.

## Review

Material and methods

Study Setting and Design

A systematic literature review was conducted according to the Preferred Reporting Items for Systematic Reviews and Meta-Analyses (PRISMA) guidelines.

Search Strategy and Study Selection

Search strings were developed and run across the electronic databases PubMed, Google Scholar, and Science Direct from inception to August 31, 2022. Studies were searched using the keywords "Monkeypox" OR "Monkeypox virus" OR "Monkeypox and travel" OR "Monkeypox outbreak" OR "Monkeypox and Africa" OR "Monkeypox and Europe".

Inclusion and Exclusion Criteria

All the studies published as full-text articles in indexed journals, involving all levels of evidence, which investigated the MPVX infection all over the world from inception, were included. Only articles published in English with available abstracts were included, with no restriction imposed in terms of the date of publication. We excluded systematic reviews and metanalysis, review articles, commentaries and correspondences, expert opinions, letters to the editor, studies on animals, unpublished reports, and book chapters.

Data Extraction and Analysis

Two authors (R.K. and S.S.) independently screened the data from the selected studies by reading the abstracts. After excluding non-eligible studies by duplicate studies and exclusion criteria, the full texts of the remaining articles were evaluated for eligibility. To minimize the risk of bias, the authors reviewed and discussed all the selected manuscripts, the references, and the articles excluded from the study. Any disagreements were resolved by consensus after consulting with a third author (S.K.S.). At the end of the process, potentially missed studies were further manually searched for among the reference lists of the included papers.

For each study included in the present review, the following data were extracted: author name, country of the report, year/period of the report, number of patients, patient demographics (e.g., age and gender), travel history, sexual history (<21 days) (gay/bisexual/MSM), contact with infected animal or person, previous smallpox vaccination, and outcome of the disease.

Data Synthesis

The study quality and characteristics of interest were tabulated and narratively described.

Ethics

As this is a systematic review using scientific articles available on public platforms, without providing any information related to patients' identities, the ethics committee's review and approval were not required.

Results

Literature Search

**Figure 1:**
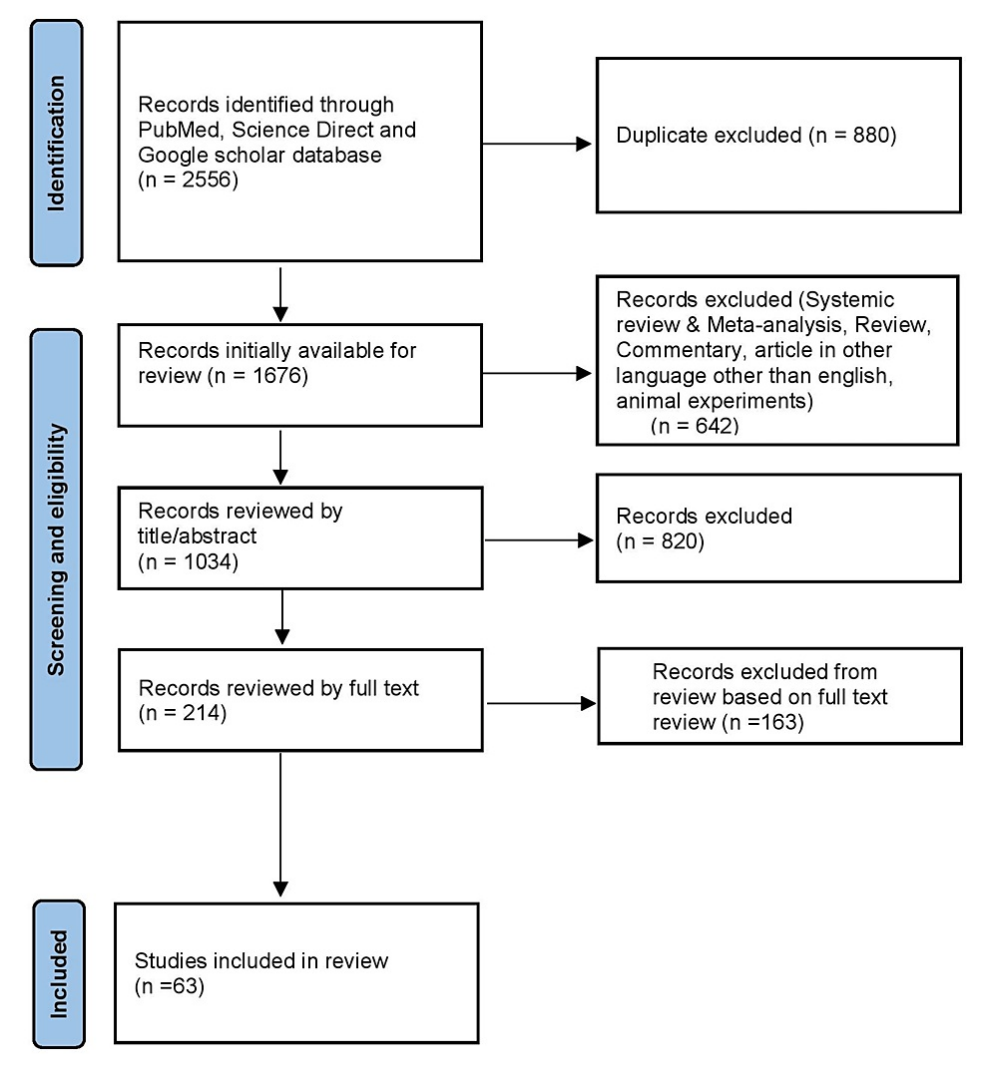
PRISMA flow chart depicting study selection for systematic review PRISMA: Preferred Reporting Items for Systematic Reviews and Meta-Analyses

Based on the eligibility criteria, 2556 studies were selected from three databases in the initial research. A total of 1676 articles were initially available for review after removing duplicate studies (880). Of these,1034 articles were selected for review after applying the inclusion and exclusion criteria. Finally, 63 articles were chosen for this systematic review after removing papers based on the evaluation of title/abstract and full text. The PRISMA flow diagram shows the process of study selection (Figure 1)

Demographic Data

A total of 5110 monkeypox cases from 63 selected articles were analyzed, including 17 case reports and 46 case series, mainly from Africa, followed by the USA, UK, Spain, and other countries (Table 1).

**Table 1:** Summary of monkeypox cases M: male; F: female; NM: not mentioned; MSM: Men Having Sex With Men; (+): yes; (-): no

Sr. no.	Author	Year/period	Country	Total cases	Male/female	Age (years)	Travel history	Sexual history (MSM/gay/bisexual)	Contact with infected animal or infected person or event attended	Previous smallpox vaccination	Outcome
1	Ortiz-Martínez Y et al. [19]	2022	USA	1	M	36	+ (contact person)	+	-	NM	Recovered
2	Pembi et al. [20]	2022	Nigeria	1	M	30	-	-	+/-	-	Recovered
3	Noe et al. [21]	2022	Germany	2	M	26, 32	-	+ (2)	-	NM	Recovered
4	Patel et al. [22]	2022	UK	197	M (197)	Median age: 38 (21-67)	+ (54)	+ (170)	+ (155) contact	NM	NM
5	Selb et al. [23]	2022	Germany	521	M (521)	Median age: 38 (20-67)	+ (70)	+ (259)	+ (151) events	NM	NM
6	Iñigo Martínez et al. [24]	2022	Spain (Madrid)	508	M (503); F (5)	Median age: 35 (18-67)	+ (38)	+ (427)	+ (72) pets + (289) contact + (73) events	NM	NM
7	Peiró-Mestres et al. [25]	2022	Spain (Barcelona)	12	M (12)	Median age: 38.5 (32-52)	+ (4)	+ (12)	+ (5) event + (4) confirmed case	+ (4)	NM
8	Jang et al. [26]	2022	Korea	1	M	34	+ (Germany)	-	+ (suspected case)	NM	NM
9	Yang et al. [27]	2022	Taiwan	1	M	20	+ (Germany)	-	NM	-	NM
10	Girometti et al. [28]	2022	UK	54	M (54)	Median age: 41	+ (25)	+ (54)	+ (2)	NM	Recovered
11	Orviz et al. [29]	2022	Spain (Madrid)	48	M (48)	Median age: 35	-	+ (42)	+ (7)	+ (12)	Recovered
12	Tutu van Furth et al. [30]	2022	Netherlands	1	M	10	+ (Turkey)	-	NM	-	Recovered
13	Oprea et al. [31]	2022	Romania	1	M	26	-	-	-	-	Recovered
14	Claro et al. [32]	2022	Brazil	1	M	41	+ (Europe)	-	-	-	NM
15	Miura et al. [33]	2022	Netherlands	18	M (18)	23-64	-	+	-	-	NM
16	Mileto et al. [34]	2022	Italy	1	M	33	+ (Portugal)	+	-	-	Recovered
17	Patrocinio‑Jesus et al.[35]	2022	Portugal	1	M	31	-	+	-	-	Recovered
18	Minhaj et al. [36]	2022	USA	17	M (17)	28-61, average age: 40	+ (14)	+ (16)	-	NM	NM
19	Ferraro et al. [37]	2022	Italy	29	M (26); F (1)	20-54, median age: 36	+ (23)	+ (16)		NM	NM
20	Bížová et al. [38]	2022	Czech Republic	1	M	34	+ (Europe)	+	-	NM	NM
21	Antinori et al. [39]	2022	Italy	4	M (4)	30s	+ (4)	+ (4)		NM	Recovered
22	Hammerschlag et al. [40]	2022	Australia	1	M	30	+ (Europe)	+	-	NM	Recovered
23	Vivancos et al. [41]	2022	UK	86	M (79); F (7)	Median age: 38	+ (1) Nigeria	+ (66)	-	NM	NM
24	Perez Duque et al. [42]	2022	Portugal	27	M (27)	22-51, median age: 33	+ (4)	+ (18)	+ cat (2), pigs (1), confirmed case (1)	+ (1)	Recovered
25	Rao et al. [43]	2021	USA	1	M	Middle-aged	+ (Nigeria)	-	-	-	Recovered
26	Hobson et al. [10]	2021	UK	3	M (3)	18 months child, adult (2)	+ (Nigeria)	-	-	NM	Recovered
27	Costello et al. [44]	2021	USA	1	M	28	+ (Nigeria)	-	-	-	Recovered
28	Kyaw et al. [45]	2019	Singapore	1	M	38	+ (Nigeria)	-	+	NM	NM
29	Eseigbe et al. [46]	2018	Nigeria	2	M (2)	20, 20	-	-	NM	NM	Recovered
30	Erez et al. [9]	2018	Israel	1	M	38	+ (Nigeria)	-	+	NM	Recovered
31	Besombes et al. [47]	2018	CAR	6	F (6)	7-33 (4), 4, 5M	-	-	+	-	NM
32	Vaughan et al. [48]	2018	UK	2	M (2)	NM	+ (2) (Nigeria)	-	+	NM	Recovered
33	Ogoina et al. [49]	2017	Nigeria	1	M	34	NM	NM	NM	NM	Suicide
34	Ogoina et al. [50]	2017	Nigeria	21	M (17); F (4)	6-45, median age: 29	-	NM	NM	NM	Recovered
35	Doshi et al. [51]	2017	Congo	22	M (8); F (14)	1-40, median age: 11.5	-	-	+	NM	Death (3) (4, 14, 40Y)
36	Yinka-Ogunleye et al. [52]	2017-2018	Nigeria	122	M (84); F (38)	2D-40Y, median age: 29	-	-	+	NM	Death (7), mean age: 27Y
37	Ogoina et al. [53]	2017-2018	Nigeria	40	M (31); F (9)	<35Y=67.5%, 28D-54Y	-	-	NM	NM	Death (5) (28D, 27, 34, 42, 43Y), anxiety and depression (11)
38	kalthan et al. [54]	2016	CAR	26	M (14); F (8)	<30=69.3%, median age: 24	-	-	+	+5 (19.2%)	Death (2) index case (36Y), child (12M)
39	Eltvedt et al. [55]	2016	Congo	1	M	4	-	-	NM	-	Death
40	Nakoune et al. [56]	2015-2016	CAR	10	M (6); F (4)	15M- 41Y	-	-	+	NM	Death (2) (15M, 5Y old)
41	Kalthan et al. [57]	2015	CAR	12	M (6); F (6)	Average age: 25	-	-	+	NM	Fatality was 67% among children less than 10 years
42	Whitehouse et al. [58]	2011-2015	Congo	1057	M (568); F (486)	1M-79Y, median age: 14	-	-	+	+ (97)	Death (8)
43	Reynolds et al. [59]	2014	Sierra Leone	2	M (2)	11M, 35Y	-	-	+	NM	Recovered
44	McCollum et al. [60]	2011-2014	North and South Kivu of Congo	3	M (2); F (1)	23-28	-	-	+	-	NM
45	Nolen et al. [61]	2013	Congo	63	M (36); F (27)	Median age: 10, 4M-68Y	-	-	NM	+ (9)	Death (10)
46	Mbala et al. [62]	2007-2011	Congo	4	F (4) Pregnant	20-29	-	-	+	NM	Miscarriage in the first trimester (2), Foetal death in 18 wks
47	Reynolds et al. [63]	2010	Congo	2	F (2)	7, 16	-	-	+	NM	NM
48	Doshi et al. [64]	2005-2007	Congo	223	M (155); F (68)	<14 (152), >30 (14)	-	-	+ (165)	+ (8)	NM
49	Rimoin et al. [65]	2005-2007	Sankuru District, Congo	760	M (472); F (288)	Average age: 11.9, 5D-70Y	-	-	+	+ (29)	NM
50	Formenty et al. [66]	2005-2006	Sudan	19	M (9); F (10)	8M-32Y, <20Y=15	-	-	NM	NM	No death
51	Rimoin et al. [67]	2001-2004	Congo	51	M (28); F (23)	Average age: 10, <24=48	-	-	+	NM	NM
52	Boumandouki et al. [68]	2003	Congo	8	M (4); F (4)	Mean age: 9.05, 5M-18Y	-	-	+	NM	Recovered
53	Reynolds et al. [69]	2003	USA	30	M (13); F (17)	<18 (10)		-	+ prairie dog (30), rodents (4)	+ (6)	Recovered
54	Huhn et al. [70]	2003	USA	34	M (18); F (16)	>18 (24)	-	-	+ home pet (19) pet store (4), veterinarian office (9)	+ (7)	Admitted to ICU <18Y, pediatrics (50%), adult (9%)
55	Sejvar et al. [71]	2003	USA	3	M (1); F (2)	6, 30, 33	-	-	+ prairie dogs	+ (1)	Child required hospitalization for severe encephalitis
56	CDC update [72]	2003	USA	35	M (17); F (18)	<18 (11), 6-51	-	-	+ prairie dog and human (30)	+ (8)	Two children had a serious clinical illness
57	Meyer et al. [73]	2001	Congo	23	NM	1-30	-	-	+	NM	Death (5) (1.5-14Y)
58	Meyer et al. [74]	2001	Gabon	4	Children (4)	NM	-	-	+	NM	Fatal case (2), hemorrhagic fever
59	Hutin et al. [75]	1996-1997	Katako-Kombe (Congo)	88	M (50); F (38)	Median age: 10, 1m-62Y	-	-	+	+ (13)	Death (3), 3.7% case fatality rate, all are children <3Y
60	CDC update Congo [76]	1996-1997	Kasai (Congo)	419	NM	<16 (357)	-	-	+ (53% another case-patient)	+ (20)	Death (5), 4-8Y
61	Heymann et al. [77]	1981-1986	Congo	338	M (183); F (155)	<10 (291), 3M-69Y	-	-	+ (245)	+ (13)	Death (10%)
62	Jezek et al. [78]	1981-1985	Zaire (Congo)	91	NM	<15 (93%), 7M-29Y	-	-	+ animal (70), human (21)	+ (10)	Death (9%)
63	Breman et al. [79]	1970-1979	Central and West Africa	47	M (26); F (21)	Mean age: 8, 7M-35Y, <10 (83%)	-	-	+	+ (4)	Death 8 (17%), 7M-7Y old

The median age of cases was 20 years in the earlier outbreaks; however, in the current outbreak, the infection occurred more frequently in the adult population (Table 1). The combined data of all studies showed that MPVX infection has continued to occur more frequently in males (64.22%), uniformly across all outbreaks. Moreover, there were almost 1519 (99.02%) male cases in the current epidemic out of the 1534 evaluated cases (Table 2).

**Table 2:** Demographic and clinical profile of patients with monkeypox (1970-2022) MSM: Men Having Sex With Men

Variables	Year/years	N (%)
Total number of monkeypox cases	1970-2022 (all studies)	5110 patients
Sex	1970-2022 (all studies)	Male: 3282/5110 (64.22%)
		Female: 1382/5110 (27.04%)
		Not mentioned: 446/5110 (8.74%)
	2022	Male: 1519/1534 (99.02%)
		Female: 13/1534 (0.98%)
Travel history	2018-2022	Yes: 252/1551 (16.24%)
Sexual history (<21 days) (MSM/gay/bisexual)	2022	Yes: 1134/1534 (73.92%)
Contact with infected animal or infected person or attended events	1970-2002	Yes (720/990) (72.72%)
	2003 in the USA	Yes: 102/102 (100%) with prairie dog, pet store, and infected human
	2003-2021	Yes: (2254/2464) (91.47%)
	2022	Yes: (764/1534) (49.80%)
Previous smallpox vaccination	1970-2022	Yes: 238/5110 (4.65%)
		No: 3057/5110 (59.82%)
		Not mentioned: 1815/5110 (35.53%)
Outcomes	1970-2022	Death: 107/5110 (including suicide: 1) (2.09%)
		Recovered: 2511/5110 (49.13%)
		Not mentioned: 2594/5110 (48.78%)

Vaccination With SPX Vaccine

Vaccination status was available to us for only 3295 out of 5110 evaluated cases. Of these cases, 3057 (59.82%) patients were unvaccinated (Table 2)

Outcome

We could ascertain the outcome in only 2618 out of 5110 evaluated cases. Of these 2618 MPVX cases, 2511 (49.13%) patients recovered, and 107 (2.09%) patients died; most were children below the age of 10 years. Of the 1534 evaluated cases during 2022, there was no mortality. Our results showed that children were at a greater risk of severe MPVX infection (Table 2). One study in our review reported that out of four MPVX-infected pregnant women, three females had miscarriages around the first trimester (Table 1)

Travel History

Before 2018, travel history was not well documented in the reviewed literature. Between 2018 and 2022, we evaluated 1551 cases, and 252 of these (16.24%) had a history of travel to endemic/infected areas, which could explain the source of infection.

Sexual Transmission

In 2022, 1134 out of 1534 (73.92%) patients admitted to having sexual relations within the last 21 days (MSM/gay/bisexual). Before 2022, not much data existed about the history of sexual relations (Table 2)

History of Contact With Infected Animals and Humans

Most patients confirmed contact with an infected animal or person in the earlier reviewed outbreaks:P 72.72% between 1970-2002 and 91.47% between 2003-2021. However, during the year 2022, 764 out of 1534 (49.80%) reviewed MPVX cases had a history of contact with an infected person or attended large-scale public events but hardly any contact with an infected animal (Tables 1, 2).

Discussion

This systematic review evaluated the MPVX outbreaks from 1970 to 2022 based on 5110 cases and aimed to find the contrasts between earlier outbreaks and the current one.

Our results showed that the disease was primarily seen in younger populations during previous outbreaks. The current outbreak, however, shows an increased median age of infection. A recent systematic review, which evaluated MPVX cases through the summer of 2018, published findings similar to our study and stated that the weighted median age of MPVX infection in Africa has increased from four and five years in the 1970s and 1980s to 10 and 21 years in the 2000s and 2010s [80]. This data correlated well with intensified vaccination drive in the 1970s and the cessation of routine SPX vaccination following its eradication in 1980. During the 2000s, only the population below 20 years of age was susceptible to MPVX, as the rest was already vaccinated. The rise in the median age to 21 years in the next decade can also be explained as, at that time, most cases were either too young to have been vaccinated or were not born yet. The increase in the median age of patients during the current outbreak can be due to the disease clustering in MSM. This disease clustering in MSM is likely the reason for recent cases’ significantly greater male preponderance.

Almost 60% of the patients with known vaccination status were unvaccinated in this review. Data from outbreaks within the DRC (1981-2013) and the US (2003) illustrated that 80-96% of cases occurred in unvaccinated individuals. The US outbreak revealed the highest percentage of vaccinated patients (21%) [80]. Using statistical modeling tools, researchers concluded that in 2016, the year before the Nigerian outbreak, only 9.3% of the population was vaccinated against SPX, and individual-level immunity was down to 2.2% from 65.6% in 1970. Almost 96% of monkeypox cases over the years have occurred in unvaccinated individuals [81,82]. When two viruses fight for the same host (in our case, SPX and MPVX are fighting for humans), only the virus with the higher R_0_ can win, which was SPX until 1980. Consequently, no MPVX cases were reported during the SPX era. The eradication of SPX in 1980 and the fall of orthopoxvirus immunity provided by SPX vaccination to about 10% has caused an entire generation to be susceptible to MPVX infection (R_0_ of MPVX now reaches anywhere between 1.1 and 2.4, adequate for human-to-human transmission) [83].

Overall mortality has decreased in the current outbreak compared to previous ones, possibly because children were affected more in the earlier outbreaks and because of a lack of antiviral drugs and other supportive therapies to manage the disease. Death was reported in approximately 2% of the patients for whom outcome data were available during this review. Most of these fatalities occurred in children. Fatalities have rarely been reported in outbreaks outside of Africa. Between the 1970s and 1990s, 100% of deaths were among children less than 10 years of age. Whereas from 2000 to 2019, only 37% of deaths occurred in children <10 years of age [80]. During this review, we also found an increased tendency among children with MPVX to have a severe illness. Studies have reported respiratory complications, sepsis, bacterial superinfection, and encephalitis more frequently in children and the immunocompromised. These complications have increased children's mortality and hospitalization rates even in high-income countries [72,84]. The CDC recommends that children with MPVX be ideally cared for in a healthcare facility by a parent or caregiver at low risk of contracting the disease until symptoms subside. The antiviral drug tecovirimat might be considered in the pediatric age group of above two years, considering the morbidity associated with MPVX in children.

Our study also reviewed fetal outcomes in four pregnant women. Only one of the four infected women (with mild disease) in a cohort of 222 symptomatic human-MPVX cases gave birth to a healthy infant devoid of MPVX symptoms. Unfortunately, two women in their first trimester of pregnancy, with moderate and severe infection, respectively, miscarried. One woman who was also coinfected with malaria gave birth to a stillborn, macerated fetus with characteristic lesions of MPVX [62]. In 1988, Jezek et al. also reported a case of a pregnant female with MPVX infection who gave birth to a premature infant with a skin rash suggestive of MPVX at 24 weeks of gestation. The child died six weeks later of unknown causes [85]. Data from the historical SPX infections also suggests that the disease is more severe in pregnant females, especially in their third trimester [86-91]. We need to generate more data regarding maternal and fetal outcomes in human-MPVX cases, factors affecting outcomes in pregnancy, the crucial time for transmission, prophylactic and therapeutic strategies, and breastfeeding status. This is important, considering that pregnancy is a state of being immunocompromised when chances of infections are higher.

Publications about the monkeypox outbreak in the DRC during the 1980s have reported the possibility of an animal source in 72.5% (245/338) of cases and a human source in 27.5% (93/338) of patients, a finding also corroborated in our study. During the 1990s, however, 22% of cases reported no contact with a human subject. In contrast, the rest did have evidence of contact with an infected person within 7-21 days before the onset of the disease. In the Nigerian outbreak (2017-2018), 78.3% of the cases for whom transmission details were known had a history of contact with people having MPVX-like lesions. The rest reported contact with animals [80].

Our study also found that approximately 50% of cases had a history of contact with an infected person or attended a super spreader (International Pride Event on Gran Canaria and other public gatherings) event during the year 2022. About 74% of patients reported having sexual relations within the last 21 days (MSM/gay/bisexual) during the year 2022. A prospective cross-sectional study of MPVX cases in 2022 in Spain also concluded that contact during sex and MSM are strongly associated with infections. Of note, 76% of patients in the study had sexually transmitted diseases [92]. The MPVX predominantly affects men, a finding reiterated in our research. The combined data in our study showed that among H-MPVX cases for whom gender data was available, almost 64% were men. This male predominance might be linked to the virus finding a favorable transmission mode through tightly interconnected sexual networks in the MSM/gay/bisexual community. Here the virus can spread in ways that it cannot in the general population. Although how the virus exactly travels in this community is unclear. It might be that skin-to-skin contact is enough like it is for other STIs. Herein might lie the most crucial factor leading to a make-or-break situation during the current outbreak. Protecting those most at risk and limiting the spread are both interlinked.

There has been much debate about the reasons for the resurgence of MPVX, with waning immunity to SPX vaccination, genetic evolution of the virus (a gene loss that correlates with human-to-human transmission), improved adaptation to the human host, a super spreader event, population growth, increasing global connectedness, deforestation and changes in land usage patterns emerging as the main culprits.

Strengths and Limitations

This is an important study summarizing MPVX outbreaks from 1970 to 2022. However, the uniformity and content of published literature varied considerably, which might have restricted the thoroughness and homogeneity of the evaluated literature. Some of the cases might be duplicated across published papers.

Recommendations

Some key points must be remembered for optimal control of the outbreak. The case fatality rate with human-MPVX in African countries has ranged from 0-11%, the highest being among young children. Evidence suggests that SPX vaccination also protected against Monkeypox (85%). However, the end of routine SPX vaccinations in 1980, which had offered cross-protective immunity against MPVX, has increased the susceptibility of people younger than 50 years to monkeypox. Children with MPVX infections need a bit of extra vigilance and timely therapeutic intervention in hospital settings, considering the apparent absence of “herd immunity” and the severity of illness in this age group. A high index of suspicion in pregnant females who present with a rash and lymphadenopathy is advised. The current outbreak is mainly confined to MSM with high-risk factors for STDs, although this trend might change anytime. Appropriate media campaigns encouraging these groups to freely come forward for testing and treatment without fear of social alienation and simultaneously avoid high-risk behavior need to be started. Containment efforts must be instituted aggressively through identification, isolation, information, contact tracing, ring vaccination (contacts of the index case and high-risk groups), and post-exposure vaccination. Mass immunization might be an option in areas where MPVX becomes endemic. Social media campaigns sharing essential information about monkeypox transmission, treatment, and vaccination protocol can decrease the impact of misinformation. The MPVX virus is unlikely to cause a significant problem except in resource-limited settings, mainly because vaccines and drugs are available, and disease manifestations are easy to identify. There is little need for a COVID-19-like panic currently or in the future.

## Conclusions

The geographical spread of MPVX has now become a matter of global concern. This systematic study has reviewed both the past and current outbreaks, concluding that the clinical picture of MPVX is changing. Awareness of evolving clinical manifestations is mandatory for healthcare professionals so that human-MPVX cases can be identified, isolated, and treated correctly. A rise in human-to-human transmission, as witnessed in the current outbreak, puts healthcare professionals and caregivers at risk, necessitating adequate preventive measures to prevent the spread. Mutations within the viral genome and an increasing R_0 _of the virus might make future outbreaks extremely difficult to control and manage, especially when transmission modes have also changed. Epidemiological investigations must be at an all-time high to assimilate all this new data and arrive at valid conclusions. The case definitions need to be reviewed, and routes of viral transmission better understood to define infection control policies as well as prevention and management strategies.
